# T Cell Memory to Vaccination

**DOI:** 10.3390/vaccines6040084

**Published:** 2018-12-14

**Authors:** Stephen M. Todryk

**Affiliations:** 1 Department of Applied Sciences, Faculty of Health & Life Sciences, Northumbria University, Newcastle upon Tyne NE1 8ST, UK; stephen.todryk@northumbria.ac.uk; 2 Institute of Cellular Medicine, Newcastle University, Newcastle upon Tyne NE2 4HH, UK

**Keywords:** T cells, vaccines, memory, effectors

## Abstract

Most immune responses associated with vaccination are controlled by specific T cells of a CD4^+^ helper phenotype which mediate the generation of effector antibodies, cytotoxic T lymphocytes (CTLs), or the activation of innate immune effector cells. A rapidly growing understanding of the generation, maintenance, activity, and measurement of such T cells is leading to vaccination strategies with greater efficacy and potentially greater microbial coverage.

## 1. Introduction 

Because it is a critical facet of vaccination, the continuing progress in the understanding of T cell memory is enhancing the further development of efficacious, licensed vaccines. This Special Issue of *Vaccines* is aimed at highlighting ways in which T cell memory may impact the immunogenicity, and ultimately the efficacy, of vaccines that are already in use or in development. This editorial briefly signposts some key concepts surrounding T helper (Th) memory cells without attempting to review the literature, as this has been done elsewhere [1,2]. Many vaccines in use today continue to rely on old manufacturing technology and development, and may be far from optimal in their immunogenicity and efficacy. The primary targets for vaccination among the human population tend to be either quite young or elderly and therefore suffer deficiencies in immune responses, such as immaturity in infants and exhaustion in the elderly. These groups are likely to require vaccines and vaccination regimens that efficiently deliver frequent, sustained, high doses of antigen, together with immune activating agents such as adjuvants or live organisms. Several diseases—HIV, Hepatitis C, some Flaviviruses, and Ebola virus, for example—have no licensed vaccines available to prevent or treat them. Diseases such as malaria, tuberculosis (TB), and influenza, have vaccines (Mosquirix [3], Bacillus Calmette-Guerin (BCG), and several influenza vaccines) but would benefit from improved vaccines. Antibodies have conventionally been the desired outcome of vaccination, as they are powerful effector immune responses capable of intercepting and neutralizing microbes and their components as well as instigating destructive anti-microbial innate immune responses. The measurement of specific antibody levels often provides a marker of immunogenicity and likely indicates protection from disease [4,5]. Many newer vaccines and vaccines in development are often designed to generate T cell responses that have the potential to help the antibody response, have direct effector functions themselves, or activate innate effector cells such as macrophages and neutrophils. These vaccines include conjugate vaccines, recombinant antigens with adjuvants, recombinant antigen-encoding viruses, nucleic acids, nanoparticles, and virus-like particles. Some vaccination regimens use combinations of vaccines, either administered together or at differing time points (heterologous prime-boost regimens), in order to generate optimal responses. The resulting antigen-specific T cell responses need to be of the appropriate type involving: helper T cells (Th cells, expressing cytokines and co-stimulatory molecules), and/or cytotoxic T lymphocytes (CTL), and with memory and homing capacity, and should not be exhausted or anergised via negative feedback or immune checkpoints (Table 1 and Table 2).

Ultimately, a correct formulation (antigen, vehicle, adjuvants; proportions thereof) and regimen (including the number and interval between immunizations, and route of vaccination) that generates the appropriate T cell response will be required. The measurement and characterization of these T cells, although challenging, provides useful markers of immunogenicity and efficacy, and informs on mechanisms for further vaccine development. There may or may not be a need for vaccines to emulate T cell responses generated in natural protective immunity, particularly when selecting sequences in antigens that are prone to mutation or variability for inclusion in novel vaccines, as is the case for HIV and influenza. Innate immunity also impinges on the vaccine-induced priming, boosting, and effector processes [6]. Effector lymphocytes with invariant T cell receptors or innate lymphoid cells and natural killer (NK) cells are likely to be involved. Indeed, it has been shown that innate immune memory does exist [7], the manipulation of which could be incorporated into vaccines. Finally, factors (other than age) associated with the target population for vaccination are important to consider. These factors include comorbidities, geographic factors (including microbial endemicity), nutritional status, and iatrogenic immune suppression. Thus, a highly multi-faceted approach to vaccine design is required, particularly for the currently non-tractable pathogens.

## 2. Human T Cell Effector and Memory Phenotypes

T cell memory can be defined as the collective reactivity of a population of T cells that respond to a cognate antigenic challenge—an antigen that is recognized by their T cell receptors (TcRs). This response occurs some time after the initial antigen exposure, by proliferating and/or expressing molecules that are able to mediate an effector reaction. In vivo, meaningful memory equates to protection from infection and/or disease when challenged with an infectious microbial pathogen, either naturally or experimentally. T cell memory follows initial antigenic exposure and priming, where naïve T cells (T_N_) respond to antigenic peptides complexed to major histocompatibility complex (MHC) molecules on antigen-presenting dendritic cells (DCs). The context in which the DCs encounter the antigen is imparted through their detection of vaccine-associated signals, e.g., toll-like receptor (TLR) ligands, which condition the DCs to express molecules such as IL-12. These molecules influence the phenotype of the T cells, e.g., Th1 cells, when recognizing the presented antigen within secondary lymphoid tissues (SLT). The DCs must ligate co-stimulatory molecule pairings, e.g., B7:CD28, that provide further signals to the T cells. This mediates their proliferation and differentiation into Th phenotypes (Table 1). Thus, T cells are programmed to possess particular phenotypes that allow them to address the microbial challenges they are specifically designed to combat, both antigenically and phenotypically. The phenotype is programmed epigenetically in the resulting T cells through the expression of transcription factors, which are retained during homeostatic non-antigenic-driven proliferation, controlled by growth factors, such as IL-2, IL-7 and IL-15, within anatomical niches. In addition to their specificity and phenotype, T cells often need to retain surface receptors that promote their homing to regions where they were primed and are needed, such as the mucosa (CCR6), the skin (CCR10), or the B cell region of SLT (CXCR5). Along with their effector phenotype, T cells can be identified on the basis of their memory status (Table 2). CD45RA^+^ T_N_ cells possess molecules that facilitate their entry into SLT, namely, CD62L and CCR7, where they become primed by cognate-antigen-presenting and activated DCs. After being primed, they proliferate to become effector-memory T cells (T_EM_), losing the molecules for SLT retention and CD45RA in the process. The T_EM_ circulate within the peripheral blood mononuclear cells (PBMCs) and have the ability to rapidly respond (sometimes within minutes) to the previously encountered antigens within inflamed tissues. During antigenic recall, e.g., booster vaccination, Th cells demonstrate some plasticity in their effector phenotype and can be redirected [8,9]. Once these cells respond, memory T cells—particularly CD8^+^—regain CD45RA, becoming T_EMRA_/T_E_, and secrete effector molecules. These effector molecules are particularly efficacious against intracellular pathogens with short incubation periods and fast reproduction (mainly viruses). These T cells upregulate molecules such as PD-1 and CD95, which makes them prone to inhibition and apoptosis, respectively. The purpose of this is to maintain non-inflammatory cellular homeostasis once the threat (and associated antigen, if indeed it is possible [10]) has subsided. Some of the T_EM_ become resident T memory cells (T_RM_) within the tissues [11,12] where the antigen is encountered and are again poised to rapidly respond to a challenge, although their life spans may be short. T_RM_ are kept in tissues by epithelial tissue-binding CD103, and CD69 which antagonizes the migratory function of S1PR1 in T cells and allows lymphoid memory deposits to persist. “Prime-and-trap” vaccines are being developed to specifically target T_RM_ generation [13]. A proportion of the primed cells become central memory T cells (T_CM_), which re-express the SLT-homing receptors. These cells provide a population of circulating quiescent cells that can respond to the re-encounter with an antigen within activated SLT by proliferating and differentiating into T_EM_ and T_E_ cells over the course of some days. These T_CM_ are therefore considered better suited to protect against pathogens with longer incubation periods, such as certain parasites [14]. Some T cells take on stem cell-like features (T_SCM_) to provide very long-lived memory, often residing in the bone marrow [15]. Within the SLT, follicular T helper cells (T_FH_) are generated. These cells are attracted to B cells via CXCR5 and help B cells to produce class-switched and high-affinity antibodies specific to their mutual cognate antigen through ligation via OX40, CD40L, and ICOS, as well as through secretion of IL-21 [16,17]. The B cells act as the antigen-presenting cells (APCs) for the cognate T_SCM_ by the uptake of an antigen bound to their surface antibodies. Individuals with particular immunodeficiencies provide insight into the importance of such T cell help in generating effective antibodies and protection from infectious diseases [18].

## 3. Measurement of Vaccine-Associated T Cells

The ability to measure memory T cells in association with vaccination [19] is important to establish a vaccine’s immunogenicity and may be a biomarker of efficacy by having a positive association with protection from infection and/or disease [3,4]. Important factors in the measurement of T cell responses are the methods of measurement, when to make these measurements, and in which locations. T cell responses measured in the circulating PBMCs during a vaccination regimen tend to follow the typical pattern of adaptive immune response, i.e., an initial exposure is followed by a lag phase, then a peak in the response (such as an antigen-specific IFNγ response) at about one to two weeks, that eventually settles back down to a response raised over the naïve response. Due to memory generated by the priming, a second exposure through boosting gives a more rapid, greater response. Through T_E_ attrition [20], this response then settles to a level higher than it was before the boost. Thus, the aim of boosting is to cause the T cells to reach a putative “protective” level. To date, few direct correlations between T cell responses and degrees of protection from infection and disease have been established for clinical use. “Quantiferon^TM^” and ELIspot tests have been able to detect latent *Mycobacterium tuberculosis* infection in at-risk individuals through the detection of IFNγ secretion by peripheral T cells reactive to particular TB antigens. However, such responses following vaccination for TB have not been associated with protection from infection [21], although a new vaccine has recently shown efficacy [22]. Even if the PBMC may not be the ideal location to detect the reactive T cells that need to act in specific tissues (such as the mucosa), precursors in transit (such as T_EM_ and T_CM_) are measurable with specialized or modified techniques. Ex vivo techniques on whole blood or PBMC involve the exposure to the vaccine antigen and the measurement of responses, which typically occur within one day. The most common of such tests involves bulk cytokine secretion from blood cells (whole blood assay/ELISA) and cytokine measurement by flow cytometry and enzyme-linked immunospot (ELISpot). This is done to identify responding cells at the single cell level. The expansion in the number of available flow cytometry parameters means that cells can be identified as secreting or expressing a multitude of molecules using single-cell mass cytometry and RNA sequencing, thus allowing their characterization within effector phenotypes, memory phenotypes (Table 1 and Table 2), and beyond. Molecular signatures in blood that are associated with vaccination continue to implicate T cells in protection [23]. Although it is more cumbersome, incorporating a period of culture of blood cells with antigens and other factors allows specific memory cells like T_CM_ to be revealed, as the culture promotes differentiation to T_EM_ and/or T_E_. One approach with whole blood that was cultured together with a precise vaccine formulation (antigen in adjuvant + TLR ligand) [24] revealed significantly higher T cell cytokine secretion. These results suggest that components of the whole blood interact with components of the vaccine to promote T cell reactivation in a way that might emulate in vivo events. Such an assay is capable of monitoring vaccine formulations for potency as well as testing vaccine recipients for their potential to respond to a vaccine in vivo. Being able to emulate ectopic lymphoid structures (memory depots) may provide one method of recreating and studying vaccine responses in vitro.

## 4. Conclusions

Technological advances in methods for studying memory T cells and in the design of vaccines are incrementally establishing vaccines that are capable of generating immune responses and protection from infection and/or disease against all significant infectious pathogens.

## Figures and Tables

**Table 1:** Effector T cell phenotypes (canonical markers, not exhaustive or mutually exclusive).

Features		T_h1_	T_h2_	T_h17_	T_Fh_	T_reg_	T_h22_	CTL
Secreted Molecules: (function)		IFNγ, IL-2, TNFα	IL-4, IL-5, IL-13, IL-9	IL-17 A/F	IL-21	+/-IL-10, TGFβ	IL-22	IFNγ, lytic enzymes
	Microbial Target/location:	intra-MΦ	parasites	extracellular	all	-	epithelial	intracellular
CXCR3 (inflammation)		+						+
CCR4 (CC chemokines)			+	+/−				
CCR6 (mucosal)				+				
CCR5 (inflammation)		+						
CD161 (NK cell receptor)				+				
CCR10 (skin homing)							+	
CD25 (IL-2Rα)						+hi		
CXCR5 (B cell homing)					+			
PD-1 (inhibitory)					+			
Others					ICOS, CD40L, OX40	TIM-3, LAG-3		
Transcription factors		Tbet, STAT1/4	GATA-3, STAT6	RORγt, STAT3	Bcl-6	FoxP3, STAT5	AHR	Eomes, RUNX3

R in a name in “Features” indicates “receptor” for chemokine, controlling homing; MΦ: macrophage; T_h_: Helper T cell; T_reg_: regulatory T cell; CTL: cytotoxic T lymphocyte; T_Fh_: follicular helper cell.

**Table 2:** Memory T cells (canonical markers, not exhaustive or mutually exclusive).

Markers	Function	T_N_	T_EM_	T_EMRA_/T_E_	T_CM_	T_SCM_	T_RM_	Exhausted T Cell
CD45RA	signalling	+	−	+	−	+		+/−
CD45RO	signalling	−	+	−	+	−		+/−
CD62L	homing	+	−lo	−lo	+	+		−
IL-7Ra (CD127)	proliferation	+	+/−	−	+hi	+		−
CD95	cell death	−	+hi	+hi	+hi	+		+
CCR7	homing	+hi	−lo	−lo	+hi	+		−
CD103 (αE)	epithelial homing	−	+				+	
CD69	activation	−	+/−	+	−	−	+	
CD28	costimulation	+int	lo	−lo	+hi	+	+/−	−
CD27	costimulation	+hi	+/−	−	+	+		
CXCR3	inflammation	−	−	−	+	+	+	
CD57	differentiation	−	+/−	+	−	−		+
PD-1	inhibitory	−	+	+/−	−		+/−	+

T_N_: Naïve; T_EM_: effector-memory; T_EMRA_: RA^+^ effector memory; T_E_: effector; T_CM_: central memory; T_SCM_: stem cell memory; T_RM_: resident memory.

## References

[1] Gray J.I., Westerhof L.M., MacLeod M.K.L. (2018). The roles of resident, central and effector memory CD4 T-cells in protective immunity following infection or vaccination. Immunology.

[2] Jameson S.C., Masopust D. (2018). Understanding Subset Diversity in T Cell Memory. Immunity.

[3] Moncunill G., De Rosa S.C., Ayestaran A., Nhabomba A.J., Mpina M., Cohen K.W., Jairoce C., Rutishauser T., Campo J.J., Harezlak J. (2017). RTS,S/AS01E Malaria Vaccine Induces Memory and Polyfunctional T Cell Responses in a Pediatric African Phase III Trial. Front. Immunol..

[4] Plotkin S.A. (2010). Correlates of protection induced by vaccination. Clin. Vaccine Immunol..

[5] Plotkin S.A. (2013). Complex correlates of protection after vaccination. Clin. Infect. Dis..

[6] de Bree L.C.J., Koeken V.A.C.M., Joosten L.A.B., Aaby P., Benn C.S., van Crevel R., Netea M.G. (2018). Non-specific effects of vaccines: Current evidence and potential implications. Semin. Immunol..

[7] Mourits V.P., Wijkmans J.C., Joosten L.A., Netea M.G. (2018). Trained immunity as a novel therapeutic strategy. Curr. Opin. Pharmacol..

[8] O′Shea J.J., Paul W.E. (2010). Mechanisms underlying lineage commitment and plasticity of helper CD4^+^ T cells. Science.

[9] Sallusto F. (2016). Heterogeneity of Human CD4^+^ T Cells Against Microbes. Annu. Rev. Immunol..

[10] Zinkernagel R.M. (2018). What if protective immunity is antigen-driven and not due to so-called “memory” B and T cells?. Immunol. Rev..

[11] Wilk M.M., Mills K.H.G. (2018). CD4 T(RM) Cells Following Infection and Immunization: Implications for More Effective Vaccine Design. Front. Immunol..

[12] Takamura S. (2018). Niches for the Long-Term Maintenance of Tissue-Resident Memory T Cells. Front. Immunol..

[13] Olsen T.M., Stone B.C., Chuenchob V., Murphy S.C. (2018). Prime-and-Trap Malaria Vaccination To Generate Protective CD8(+) Liver-Resident Memory T Cells. J. Immunol..

[14] Lumsden J.M., Schwenk R.J., Rein L.E., Moris P., Janssens M., Ofori-Anyinam O., Cohen J., Kester K.E., Heppner D.G., Krzych U. (2011). Protective immunity induced with the RTS,S/AS vaccine is associated with IL-2 and TNF-α producing effector and central memory CD4 T cells. PLoS ONE.

[15] Gattinoni L., Speiser D.E., Lichterfeld M., Bonini C. (2017). T memory stem cells in health and disease. Nat. Med..

[16] Vinuesa C.G., Linterman M.A., Yu D., MacLennan I.C. (2016). Follicular Helper T Cells. Annu. Rev. Immunol..

[17] Crotty S. (2015). A brief history of T cell help to B cells. Nat. Rev. Immunol..

[18] Tangye S.G., Deenick E.K., Palendira U., Ma C.S. (2012). T cell-B cell interactions in primary immunodeficiencies. Ann. N. Y. Acad. Sci..

[19] Bowyer G., Rampling T., Powlson J., Morter R., Wright D., Hill A.V.S., Ewer K.J. (2018). Activation-induced Markers Detect Vaccine-Specific CD4^+^ T Cell Responses Not Measured by Assays Conventionally Used in Clinical Trials. Vaccines.

[20] Crespo J., Sun H., Welling T.H., Tian Z., Zou W. (2013). T cell anergy, exhaustion, senescence, and stemness in the tumor microenvironment. Curr. Opin. Immunol..

[21] Voss G., Casimiro D., Neyrolles O., Williams A., Kaufmann S.H.E., McShane H., Hatherill M., Fletcher H.A. (2018). Progress and challenges in TB vaccine development. F1000Research.

[22] Van Der Meeren O., Hatherill M., Nduba V., Wilkinson R.J., Muyoyeta M., Van Brakel E., Ayles H.M., Henostroza G., Thienemann F., Scriba T.J. (2018). Phase 2b Controlled Trial of M72/AS01(E) Vaccine to Prevent Tuberculosis. N. Engl. J. Med..

[23] Haks M.C., Bottazzi B., Cecchinato V., De Gregorio C., Del Giudice G., Kaufmann S.H.E., Lanzavecchia A., Lewis D.J.M., Maertzdorf J., Mantovani A. (2017). Molecular Signatures of Immunity and Immunogenicity in Infection and Vaccination. Front. Immunol..

[24] Hakimi J., Azizi A., Ausar S.F., Todryk S.M., Rahman N., Brookes R.H. (2017). An adjuvant-modulated vaccine response in human whole blood. Hum. Vaccin. Immunother..

